# The Role of the *Fusarium oxysporum* FTF2 Transcription Factor in Host Colonization and Virulence in Common Bean Plants (*Phaseolus vulgaris* L.)

**DOI:** 10.3390/pathogens12030380

**Published:** 2023-02-26

**Authors:** Virginia Casado-del Castillo, Ernesto P. Benito, José María Díaz-Mínguez

**Affiliations:** Instituto de Investigación en Agrobiotecnología (CIALE), Departamento de Microbiología y Genética, Universidad de Salamanca, C/Río Duero, 12, Villamayor, 37185 Salamanca, Spain

**Keywords:** *Fusarium oxysporum*, virulence, *FTF* gene family, plant colonization, effector

## Abstract

The *FTF* (*Fusarium Transcription Factor*) gene family is composed of two members (*FTF1* and *FTF2*) with high-sequence homology that encode transcription factors involved in the modulation of virulence in the *F. oxysporum* species complex (FOSC). While *FTF1* is a multicopy gene exclusive of highly virulent strains of FOSC and is located in the accessory genome, *FTF2* is a single-copy gene, located in the core genome, and well-conserved in all filamentous ascomycete fungi, except yeast. The involvement of *FTF1* in the colonization of the vascular system and regulation of the expression of SIX effectors has been stablished. To address the role of *FTF2*, we generated and characterized mutants defective in *FTF2* in a *F. oxysporum* f. sp. *phaseoli* weakly virulent strain and analyzed them together with the equivalent mutants formerly obtained in a highly virulent strain. The results obtained highlight a role for *FTF2* as a negative regulator of the production of macroconidia and demonstrate that it is required for full virulence and the positive regulation of SIX effectors. In addition, gene expression analyses provided compelling evidence that *FTF2* is involved in the regulation of hydrophobins likely required for plant colonization.

## 1. Introduction

*Fusarium oxysporum* Schlechtend.:Fr. is an anamorphic species complex comprising saprophytes and plant colonizers. The second group of strains may live inside the plant roots as endophytes or infect and cause disease in many important crops. However, the pathogenic individual isolates generally are able to infect only a single species, or a small group of them, which allows us to classify them into host-specific forms known as formae speciales [1]. *Fusarium oxysporum* Schlechtend.:Fr. f. sp. *phaseoli* J.B. Kendrich and W.C. Snyder is the causal agent of Fusarium wilt in common bean plants (*Phaseolus vulgaris* L.), one of the most important diseases that reduces the production of dry beans.

The wide pathogenic ability of *F. oxysporum* as a species complex, but restricted specificity at the strain level, is surprising in comparison with other species of the genus *Fusarium*, such as *Fusarium graminearum* and *Fusarium verticillioides*. Recently, 106 formae speciales have been well-documented and 37 more have been reported but insufficiently documented [2]. Although *F. oxysporum* is mostly a plant colonizer, its abilities are not restricted to the vegetal kingdom. There have been described isolates able to infect *Caenorhabditis elegans* [3], mice, and humans [4]. The genetic basis responsible for this versatility probably relies on the particular architecture of the *F. oxysporum* genome. The core genome, very similar among all the formae speciales sequenced to date and also to the related species *F. graminearum* and *F. verticillioides*, encompasses 11 chromosomes that contain genes required for basic metabolism and differentiation. The accessory genome, which spans several lineage-specific chromosomes and some parts of the core-genome chromosomes, contains sophisticated genomic elements linked to pathogenicity, virulence, and host specificity [5,6]. The term “accessory” relates to the fact that the genetic elements in this genome are not required for basic saprophytic growth and development.

The horizontal transfer of whole or partial chromosomes may explain how an endophytic or saprophytic strain may become pathogenic as well as the diversification of formae speciales [7,8]. However, it is not the only mechanism as pathogenicity also may evolve independently [7]. In this case, the most plausible scenario would imply the mutation and functional diversification of preexisting genes in the core genome. To analyze this hypothesis, a good starting point is the observation of how gene families have evolved and diversified.

Several gene families involved in pathogenicity and virulence have been described in *F. oxysporum*. The two most important ones are the *SIX* gene family of effectors [9] and the *FTF* gene family of transcription factors. At present, 14 *SIX* genes have been identified in *F. oxysporum* f. sp. *lycopersici*. The corresponding proteins share the common feature of being “secreted in xylem”; however, the coding genes are not phylogenetically related. On the contrary, the *FTF* gene family is composed of a unique core-genome gene, *FTF2*, which is present in nonpathogenic and pathogenic strains of all formae speciales and encode a predicted polypeptide 1072 amino acids in length, and a variable number of *FTF1* paralogs [10]. The number of paralogs range from a single one in formae speciales *arabidopsis* and *conglutinans* up to 10 in forma specialis *lycopersici* race 2. These paralogs may be putative functional genes showing some variability in the length of the encoded proteins (from 930 to 1070–1079 amino acids) or putative nonfunctional truncated pseudogenes that show combinations of small and large deletions and premature stop codons. In most cases, they are linked to transposons, which suggests that duplication and translocation may be involved in their origin and diversification.

The *FTF1* paralogs have been shown to be important virulence factors that are only expressed in planta during host colonization [11] and regulate the expression of some, if not all, SIX effectors. The attenuation of *FTF* gene expression by means of gene silencing results in a dramatic reduction in the virulence and reduced expression of several *SIX* genes. The gene replacement of the *FTF2* gene in a highly virulent strain results in a less important reduction in virulence towards the host plant (common bean) [10].

In this work, we address the precise role of FTF2 in plant colonization, the nature of the genes regulated by this transcription factor, and the type of defensive response that its expression induces in the plant host. We also propose a model to explain how the expression of the *FTF* gene family modulates virulence in *F. oxysporum*.

## 2. Materials and Methods

### 2.1. Fungal Strains and Culture Conditions

The *F. oxysporum* f. sp. *phaseoli* wild-type strains FOP-SP1 (highly virulent) and FOP-SP4 (weakly virulent) [12,13] and mutant strains FOP-SP1Δ*FTF2*, FOP-SP4Δ*FTF2*, and SP1*PgpdA*::*FTF1* [10] were used in this study. All strains were grown as previously described [12,14]. Fungal cultures were established from frozen mycelia stored on 25% glycerol *v*/*v* at −80 °C and incubated under controlled conditions (25 °C and continuous light) for 6 (solid media) or 5 days at 120–180 rpm (liquid cultures).

### 2.2. Obtention of ΔFTF2 Mutants in the Weakly Virulent Strain FOP-SP4 of F. oxysporum f. sp. phaseoli

Plasmid p*FTF2*-KO [9] was used to obtain FOP-SP4 transformants with the *FTF2* gene (*locus* FOXG_09390 in strain 4287 of *F. oxysporum* f. sp. *lycopersici*) inactivated by gene replacement with a selectable marker (the hygromycin resistance gene *hph*). Fungal transformations were performed following the *Agrobacterium tumefaciens*-mediated transformation procedure, as previously described [11,15].

### 2.3. Sporulation, Germination, and Saprophytic Growth Assays

To test the sporulation rate of the fungal strains, 10^6^ fresh conidia were inoculated in 40 mL of PDB (Potato Dextrose Broth, Difco) and the cultures were incubated at 25 °C and 180 r.p.m. with continuous light. A total of 2 mL aliquots were taken at 3, 5, 7, and 10 days post-inoculation (dpi) and the concentration of spores was estimated with a Thoma cell counting chamber. Four independent cultures were analyzed in each biological experiment and the experiments were repeated three times.

To test the germination rate of the fungal strains, a suspension of fresh microconidia prepared at a concentration of 10^6^ spores/mL was used as the initial culture. A total of 100 µL of this suspension was placed in the center of an empty Petri dish. The plates were incubated at 25 °C with continuous light and high humidity for a maximum of 10 h. The concentration of germinated vs. non-germinated spores was estimated at 0, 2, 4, 6, 8, and 10 hpi. A spore was considered as “germinated” when the length of the germinative tube was at least the same as the non-geminated spore. Three independent cultures per time point were analyzed in each biological experiment and the experiments were repeated three times.

The saprophytic growth of the strains was tested on solid synthetic media. Minimal media [15] was amended either with 23.5 mM NaNO_3_ and different carbon sources (sucrose, mannose, or xylose) at 3% or 0.3%, or with 3% sucrose and different nitrogen sources (NaNO_3_, NaNO_2_, ammonium tartrate, or NH_4_NO_3_) at the concentrations previously described [16]. The pH of minimal media amended with 3% sucrose and 23.5 mM NaNO_3_ [16] was adjusted to 4.0, 6.0, or 8.0 when analyzing the effect of pH on the fungal growth. Three plates per media and strain were inoculated and three independent biological experiments were analyzed. Plates were incubated at 25 °C under a 16/8 h light/dark photoperiod. The diameter of the colony was measured at6 dpi. 

### 2.4. Inoculation of Common Bean Plants

The inoculation of *P. vulgaris* L. cv Blanca Riñón with conidia from *F. oxysporum* strains and transformants was performed as previously described [12]. After inoculation, the plants were transferred to 50 mL Falcon^®^ tubes filled with PGM (Plant Growth Medium) solution, covered with foil, and incubated in hydroponic cultures as previously described [17]. Inoculation assays were repeated three times in a randomized design. 

### 2.5. Confocal Laser Microscopy

Plants inoculated with FOP-SP1 wild-type and FOP-SP1Δ*FTF2* mutant strains were maintained in hydroponic cultures and examined after infection, as previously described, with some modifications [17]. Longitudinal and cross-sections were sliced from root system (1, 2, 3 dpi), root crown (5, 7 dpi), and hypocotyls (14, 21 dpi), and incubated overnight in 100% ethanol at 4 °C (ethanol was replaced if necessary). Then, sections were incubated in 10% KOH at 85 °C for 5 min. The samples were then washed 4 times with 1X PBS (Phosphate-Buffered Saline) pH 7.4, and then the staining solution (10 µg/mL WGA-FITC -W11261, Invitrogen, ThermoFisher Scientific Inc., Waltham, MA, USA; 20 µg/mL propidium iodide -P4170, Sigma-Aldrich; Darmstadt, Germany; 0.02% Tween 20 in 1X PBS pH 7.4) was added. The samples were vacuum infiltrated once for 10 min, followed by 10 times for 2 minutes each using a Savant DNA 120 SpeedVac Concentrator (ThermoFisher Scientific Inc., Waltham, MA, USA), de-stained washing was performed 4 times with 1X PBS, and stored overnight at 4 °C. Stained sections were visualized using a laser-scanning spectral confocal microscope (TCS2-SP2, Leica Microsystems, Bensheim, Germany). The images were analyzed using the software LAS-Advanced Fluorescence Lite 1.8.2 (Leica Microsystems, Benheim, Germany). 

### 2.6. Nucleic Acid Extraction and Purification

Samples of mycelium and plants for nucleic acid isolation were harvested or cut and immediately frozen at −80 °C. Genomic DNA was extracted from *F. oxysporum* mycelium according to the procedures previously described [11,12,18] and used in PCR reactions and Southern blot analysis. Southern blots were performed as previously described [10,11]. Total DNA from inoculated plants was isolated using the “mini-prep” DNA extraction method [19]. Total RNA was extracted both from mycelium and plants using the SV Total RNA Isolation System Z3105 (Promega, Madison, WI, USA), according to the manufacturer’s recommendations. RNA was finally treated with a TURBO DNA-free^TM^ Kit (AM1907, Invitrogen, ThermoFisher Scientific Inc., Waltham, MA, USA) to remove traces of DNA. The integrity of purified RNA was checked by running an aliquot in agarose gels and it was quantified using a Nanodrop Spectrophotometer (ThermoFisher Scientific Inc., Waltham, MA, USA).

### 2.7. Analysis of Gene Expression and Fungal Biomass Quantification

The analysis of gene expression and the quantification of fungal biomass were performed by means of Quantitative Reverse Transcription PCR (RT-qPCR) and qPCR, respectively. The qPCR reaction components and cycling conditions followed the recommendations of previously described protocols [17,20]. The efficiency of each pair of primers used in this work was verified prior to use in the quantifications, as previously described [20]. All the reactions were performed in a StepOnePlus^TM^ Real-Time PCR System (Applied Biosystems, Foster City, CA, USA), according to the manufacturer’s recommendations. The StepOne^TM^ Software v2.3 (Applied Biosystems, Foster City, CA, USA) was used for the analysis of the data. 

The Prime Script^TM^ RT Reagent Kit (Takara Bio Europe, St. Germain-en-Laye, France) was used in the synthesis of cDNA. The conditions of the synthesis followed previously described indications [17]. The *F. oxysporum EF1a* and common bean *actin* genes were used as endogenous reference genes. The 2^−ΔΔCt^ method [21] was selected for the calculations of relative expression levels of each gene. Samples from three different biological experiments and two independent cDNA preparations per biological experiment were obtained. Three replicas of each cDNA were analyzed to calculate the mean and standard deviation. All the primers used in RT-qPCR experiments are listed in Supplementary Table S1.

The quantification of the relative fungal DNA vs. plant DNA amount was performed according to previously described methods [20]. Three independent biological experiments were performed in a randomized design, six plants per time point and the condition assayed in each experiment were collected for the isolation of DNA, as it described above, and three replicas of each DNA were analyzed. A total of 100 ng of each DNA extracted from inoculated plants was used as a template in qPCR reactions. For the detection and quantification of fungal DNA primers *SGE1*-Fwd and *SGE1*-Rev were used to generate a fragment of the single-*locus SGE1* gene (*locus* FOXG_10510 in strain 4287 of *F. oxysporum* f. sp. *lycopersici*) (Supplementary Table S1). Primers *PR1*-Fwd and *PR1*-Rev were designed to generate a fragment of the single-*locus PR1* gene from the common bean genome (Supplementary Table S1), which was used as an endogenous gene to normalize differences in DNA template amounts. 

## 3. Results

### 3.1. Obtention of ΔFTF2 Mutants by Gene Replacement

The gene replacement of the *FTF2* allele in the FOP-SP4 weakly virulent wild type was performed using plasmid p*FTF2*-KO, as previously described [10]. The plasmid and strategy followed were designed to ensure the replacement of most of the *FTF2* region, leaving the *FTF1* paralogs unaltered and the coding region of a putative *locus* adjacent to *FTF2* (FOXG_09391). Several transformants were obtained and subjected to PCR and Southern analysis, which verified the deletion of *FTF2*, replaced by the hygromycin resistance (*hph*) maker gene (Supplementary Figure S1). Two transformants were selected for further analysis (FOP-SP4Δ*FTF2*-1 and FOP-SP4Δ*FTF2*-7), together with a confirmed ectopic transformant (FOP-SP4Ect-4). The lack of *FTF2* transcript in the selected transformants was verified by the reverse transcription quantitative polymerase chain reaction (RT-qPCR) (data not shown).

### 3.2. Functional Domains of the FTF Transcription Factors

The FTF2 transcription factor and those transcription factors encoded by the *FTF1* functional paralogs have two functional domains: (a) the Zn(II)_2_Cys_6_ binuclear cluster DNA-binding motif exclusive of fungi and first characterized in the *Saccharomyces cerevisiae* GAL4 protein [22], and (b) a “fungal specific transcription factor domain” or “Middle Homology Region” (MHR) [23], also called the “Major Homology Domain” (MHD), which is commonly associated with the Zn(II)_2_Cys_6_ domain [10,11].

**Figure 1:**
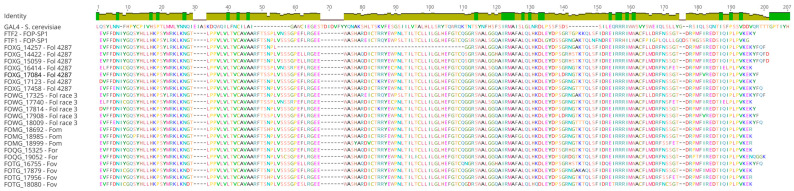
Multiple alignment of the predicted amino acid sequences of the MHD region of Gal4, FTF2, and FTF1 proteins. The corresponding *locus* code/name for the encoding gene of each protein and the species are shown in the first column. Deduced proteins from the following formae speciales were analyzed: FOP-SP1 (*F. oxysporum* f. sp. *phaseoli*); Fol 4287 (*F. oxysporum* f. sp. *lycopersici* race 2); Fol race 3 (*F. oxysporum* f. sp. *lycopersici* race 3); Fom (*F. oxysporum* f. sp. *melonis*); For (*F. oxysporum* f. sp. *raphani*); and Fov (*F. oxysporum* f. sp. *vasinfectum*).

A multiple alignment of the MHD region of 22 *FTF1* paralogs representing 5 formae speciales (7 paralogs from *F. oxysporum* f. sp. *lycopersici* race 2 strain 4287, 5 from *F. oxysporum* f. sp. *lycopersici* race 3, 3 from *F. oxysporum* f. sp. *melonis*, 2 from *F. oxysporum* f. sp. *raphani*, 4 from *F. oxysporum* f. sp. *vasinfectum*, and 1 from *F. oxysporum* f. sp. *phaseoli*) to the *FTF2* equivalent region showed a high level of conservation of the amino acidic sequence, with some diversity among the *FTF1* paralogs (Figure 1). The equivalent multiple alignment of the binuclear zinc-finger domain showed almost a complete identity (data not shown).

In order to verify the spatial differences in the domain structure, the Phyre2 web portal for protein modeling, prediction, and analysis [24] was used to compare the predicted 3D structures of the MHD domain of the 22 FTF1 paralogs and FTF2 protein. No differences that could be correlated to polymorphisms in the amino acid sequence could be detected (data not shown). The 3D structures of the binuclear zinc-finger domain were also analyzed; however, no significant differences could be observed (data not shown).

### 3.3. Phenoypic Characterization of ΔFTF2 Mutants

Δ*FTF2* mutants obtained by the gene replacement of the native *FTF2 locus* (*FOXG_09390* in the *F. oxysporum* f. sp. *lycopersici* genome) in the highly virulent wild-type strain FOP-SP1 and weakly virulent wild-type strain FOP-SP4 were subjected to in vitro phenotypic characterization by analyzing the sporulation rate, the germination rate of conidia, and the growth in synthetic media.

**Figure 2:**
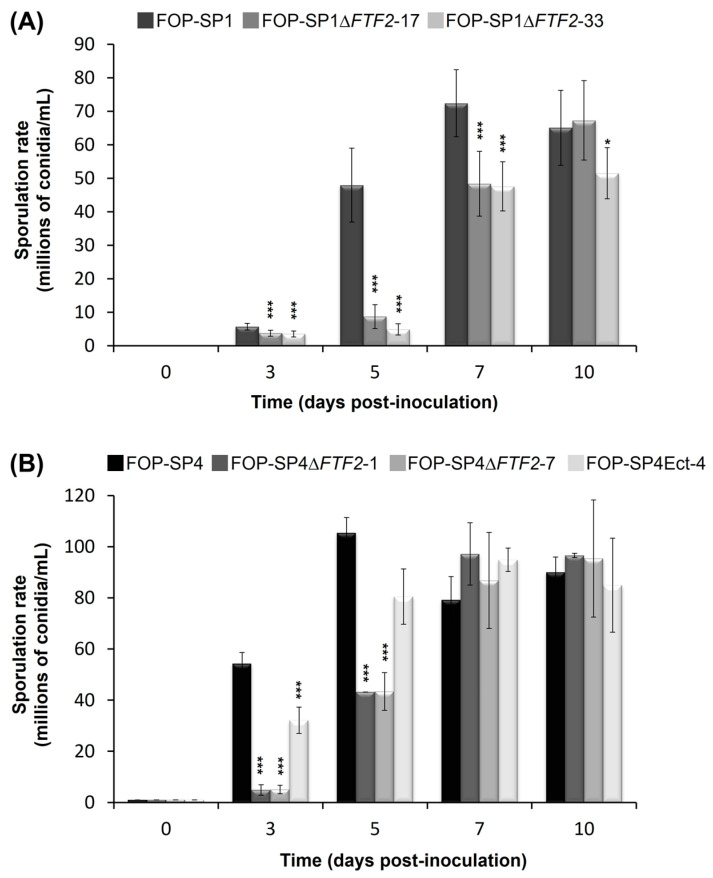
Sporulation rates of FOP-SP1Δ*FTF2* (A) and FOP-SP4Δ*FTF2* (B) strains. Mutant and wild-type strains were cultured in PDB media for a maximum period of 10 days. Each bar represents the media ± standard deviation of three independent biological experiments. Significant differences were tested using an ANOVA analysis followed by Dunnett’s test and are indicated by * (*p* < 0.05) and *** (*p* < 0.001).

**Figure 3:**
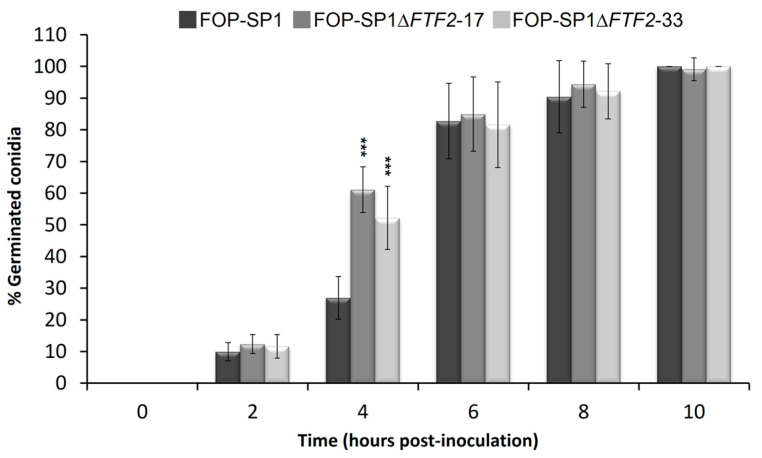
Germination rates of FOP-SP1Δ*FTF2* strains. Mutant and wild-type strains were incubated in PDB and germinated vs. non-germinated spores were analyzed for a maximum period of 10 hpi. Each bar represents the media ± standard deviation of three independent biological experiments. Significant differences were tested using an ANOVA analysis followed by Dunnett’s test and are indicated by *** (*p* < 0.001).

The sporulation rates of two independent mutants for each wild-type genetic background were determined in liquid cultures for a maximum period of ten days after inoculation (Figure 2). The mutant strains showed a delay in the sporulation rates when compared to their respective wild types. However, by 7 to 10 days after inoculation, the sporulation rates were very similar. On the contrary, germination in the mutant strains started earlier than in the wild-type strains, although the percentage of germinated spores was similar in both cases 6 h after inoculation (Figure 3; only the results for FOP-SP1Δ*FTF2* and FOP-SP1 are shown).

In order to verify the possible differences in the radial growth rate or colony morphology between the mutant strains and wild types, both groups of strains were grown on PDA and minimal medium amended either with sodium nitrate and different carbon sources at two concentrations (3% and 0.3%) or with sucrose 3% and different nitrogen sources. Additionally, growth was assayed on minimal medium amended with sucrose 3% and sodium nitrate, as the sole nitrogen source, adjusted to pH values 4.0, 6.0, and 8.0. No differences could be observed between the mutant strains and original wild types (Supplementary Figure S2).

**Figure 4:**
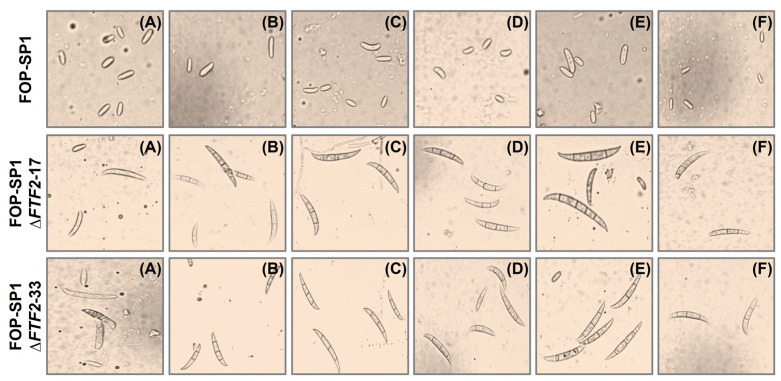
Macroconidia production by FOP-SP1Δ*FTF2* strains. Fungal strains were inoculated on synthetic solid media (PDA (**A**), and minimal medium amended with NaNO_3_ as a nitrogen source and glucose (**B**), sucrose (**C**), xylose (**D**), mannose (**E**), or glycerol (**F**) as carbon sources) under controlled conditions (25 °C and a 16/8 h light/dark photoperiod). The conidia were harvested 6 days post-inoculation. Images of a suspension of harvested conidia were taken using a Leica DC300F camera adapted to the Leica DLMB microscope (Leica Microsystems, Bensheim, Germany).

Sporulation rates were also determined for mycelia grown on solid media. Wild-type strains do not produce macroconidia, either when grown in liquid media (as previously shown) or when they are grown on synthetic solid media. However, the Δ*FTF2* mutants obtained from FOP-SP1 (FOP-SP1Δ*FTF2*-17 and FOP-SP1Δ*FTF2*-33) showed a surprisingly high production of macroconidia (Figure 4 and Supplementary Table S2), either in PDA or in some amended minimal media (with glucose, xylose, glycerol, sucrose, and mannose). Similar results were obtained for FOP-SP4Δ*FTF2* mutants (results not shown).

### 3.4. Host Plant Colonization by ΔFTF2 Mutants

One of the *FTF2* gene-replacement mutants obtained in the FOP-SP1 genetic background was used to analyze the possible changes in the in planta colonization pattern. FOP-SP1 is a highly virulent strain; therefore, the possible altered patterns of colonization determined by the absence of the FTF2 transcription factor should be more easily detected than in a weakly virulent strain. Consistent with this, infection assays conducted on common bean plants inoculated with FOP-SP4Δ*FTF2* mutants showed only a slight virulence reduction when compared to plants inoculated with the weakly virulent wild-type strain FOP-SP4 (Supplementary Figure S3).

**Figure 5:**
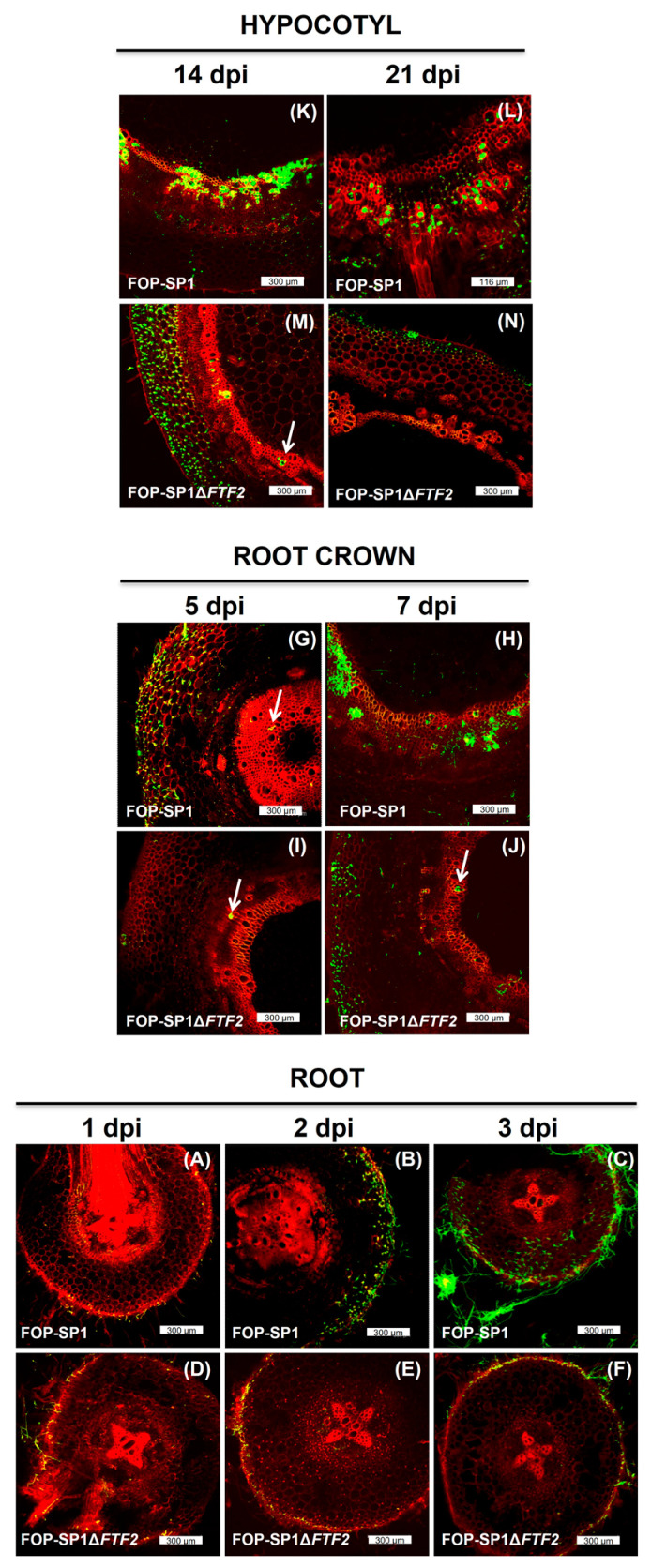
Common bean colonization by FOP-SP1ΔFTF2 mutant. Sections of plants colonized by FOP-SP1 and a selected FOP-SP1ΔFTF2 mutant were double-stained with WGA Alexa FluorTM 488 and propidium iodide. Confocal laser microscopy was used to visualize the progress of in planta growth. Sections from the root system (**A**–**F**) were sliced at 1 (**A**,**D**), 2 (**B**,**E**), and 3 (**C**,**F**) dpi, from the root crown (**G**–**J**) at 5 (**G**,**I**) and 7 (**H**,**J**) dpi, and from hypocotyls (**K**–**N**) at 14 (**K**,**M**) and 21 (**L**,**N**) dpi. Upper images in each panel show plant tissues colonized by FOP-SP1, while bottom images correspond to the FOP-SP1Δ*FTF2* mutant. White arrows show fungal growth inside vascular vessels.

Longitudinal and transversal sections of the root system, root crown, and hypocotyl of common bean plants were sampled at different times after inoculation. The characteristics and extent of fungal colonization were visualized by means of WGA-FITC and propidium iodide staining, as shown in Figure 5.

The *FTF2* mutant shows a reduced ability to colonize the root system, which is more evident three days after inoculation. Additionally, the ratio of colonized parenchymal tissue versus vascular tissue was increased at later stages, both in the root crown and hypocotyl. This colonization pattern highly resembles that displayed by weakly virulent strains [20] and mutants attenuated in the expression of the *FTF* gene family [10].

The altered pattern of colonization and drastic reduction in fungal hyphae inside the vascular system was not due to an overall reduction in the fungal biomass. The amount of fungal biomass detected by means of qPCR in plants inoculated with the wild-type and mutant strain was not significantly different (Supplementary Figure S4).

### 3.5. Plant Defensive Response

To evaluate the role of FTF2 in the induction of the plant defensive response, possible changes in the expression of selected plant genes were assessed in RT-qPCR experiments. To this point, the expression of genes involved in the salicylic (*PR1*) and ethylene/jasmonic acid (*ERF1* and *ERF2*) responses was analyzed in the root system, root crown, and hypocotyl of common bean plants inoculated with the wild-type strain (FOP-SP1) or one of the *FTF2* mutants obtained from this strain (FOP-SP1Δ*FTF2)*.

**Figure 6:**
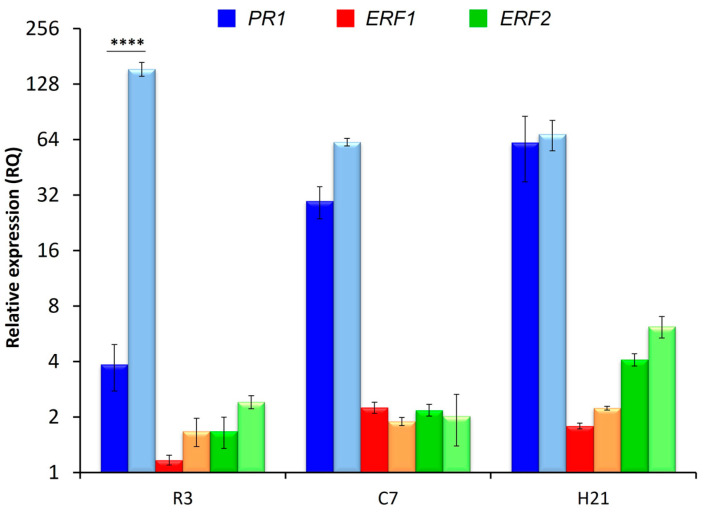
RT-qPCR analysis of expression of common bean genes involved in the defense response (*PR1*, pathogenesis response; *ERF*, ethylene response factors) in inoculated plants. The plant regions assayed and the time intervals after inoculation are indicated on the *X* axis (R3, root system 3 dpi; C7, root crown 7 dpi; H21, hypocotyls 21 dpi). The relative expression measurements on the *Y* axis are indicated in a logarithmic scale. Dark bars of each color indicate gene expression in plants colonized by FOP-SP1; light bars indicate gene expression in plants colonized by FOP-SP1Δ*FTF2*. The expression ratios were normalized by using the common bean *actin* gene as an endogenous control. The value 1.0 was denoted for the transcript level of all genes in mock inoculated plants for each plant region (data not shown). The levels of expression for a pair of measurements were tested using a *t*-test and significant differences are indicated by **** (*p* < 0.0001).

The results presented in Figure 6 show a significant increase in the expression of *PR1* in roots colonized by the mutant strain 3 days post-inoculation (dpi); although, no differences in the expression of this gene could be detected during later stages of colonization. The expressions of *ERF1* and *ERF2* in plants inoculated with the wild type were in line with the results previously obtained [20]. The lack of expression of *FTF2* did not change this expression pattern.

### 3.6. Analysis of FTF2-Responsive Genes

To shed more light on the functional role of the FTF2 transcription factor, we performed an expression analysis of several putative FTF2-responsive genes. These genes were selected from a preliminary transcriptomic comparative analysis of 48 h-old liquid cultures inoculated with FOP-SP4 and one of the *FTF2* mutants obtained from this strain. As FOP-SP4 is a weakly virulent strain, it lacks all the *FTF1* paralogous genes, thus ensuring that the differentially expressed genes obtained from the transcriptomic analysis would be exclusively the result of *FTF2* regulation.

We selected five genes: FOXG_14730 (putatively encoding a protein with SET domain), FOXG_02746 and FOXG_02748 (putatively encoding type II hydrophobins), FOXG_14731 (putatively encoding an oxygenase), and *FTF2* itself, to analyze their expressions in different genetic backgrounds, namely, both wild types (FOP-SP4 and FOP-SP1), the null mutants obtained by *FTF2* gene replacement in both strains (FOP-SP4Δ*FTF2* and FOP-SP1Δ*FTF2*), a FOP-SP4 mutant containing an ectopic integration of the construction used to replace *FTF2* (FOP-SP4Ect-4), and, finally, a mutant of FOP-SP1, which constitutively expresses one of the functional *FTF1* paralogs under the control of the *gpdA* promoter (SP1-*PgpdA::FTF1*) [10].

**Figure 7:**
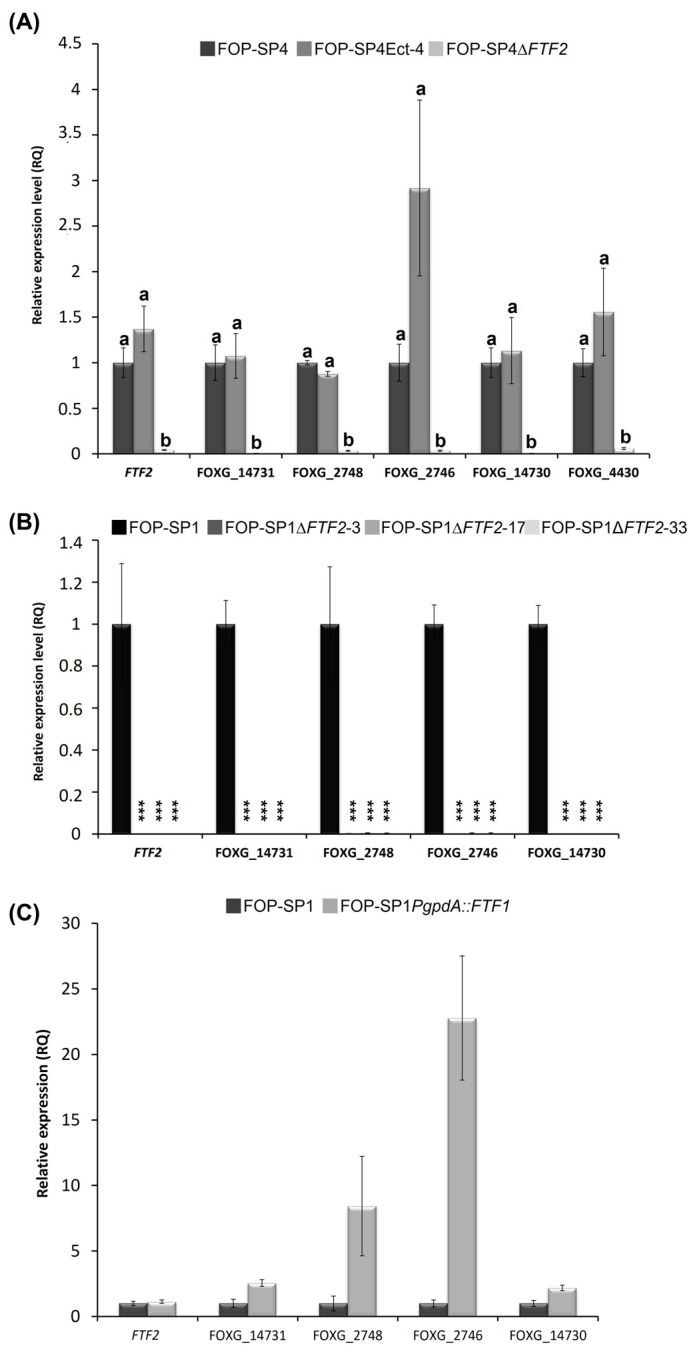
RT-qPCR analysis of expression of putative FTF2-responsive genes. (**A**) Analysis performed in FOP-SP4Δ*FTF2* strain. The value 1.0 was denoted for the transcript level of all genes in the wild-type strain FOP-SP4. Letters over each bar represent significant differences in the expression of the genes in the strains assayed after an ANOVA analysis followed by a Tukey’s HSD test (*p* < 0.05). (**B**) Analysis in FOP-SP1Δ*FTF2* strains. The value 1.0 was denoted for the transcript level of all genes in the wild-type strain FOP-SP1. Differences were tested using ANOVA analysis followed by Dunnett’s test and are indicated by *** (*p* < 0.001). (**C**) Analysis of gene expression in SP1*PgpdA*::*FTF1* strain. The value 1.0 was denoted for the transcript level of all genes in the wild-type strain FOP-SP1. In all cases, the expression ratios were normalized by using the *EF1α* gene as the endogenous control and bars represent the media ± standard deviation of three independent biological experiments.

The RT-qPCR analysis of expression in mycelia grown in PDB medium is shown in Figure 7. As expected, the expression of the five genes could not be detected in FOP-SP4Δ*FTF2* (Figure 7A). Additionally, a complete lack of expression of the five genes could be observed in FOP-SP1Δ*FTF2* mutants (Figure 7B). These results indicate that the genes analyzed are regulated by the FTF2 transcription factor. However, as there is no significant in vitro expression of the *FTF1* paralogs, it could not be ruled out that *FTF1* expression could activate the transcription of some or all of the selected genes.

To answer this question, two approaches were followed. First, a transformant that constitutively expressesed one of the *FTF1* paralogs under the control of the *gpdA* promoter (SP1*PgpdA*::*FTF1*) [10] was analyzed (Figure 7C). All the genes, except *FTF2*, showed a significant upregulation when *FTF1* was constitutively expressed. These results demonstrate that even though all the genes were selected on the basis of differential expression in the absence of *FTF2*, they also respond positively to the presence of *FTF1*. However, a high number of *FTF1* transcripts, such as those produced by the strong *gpdA* promoter, is required for this activation.

**Figure 8:**
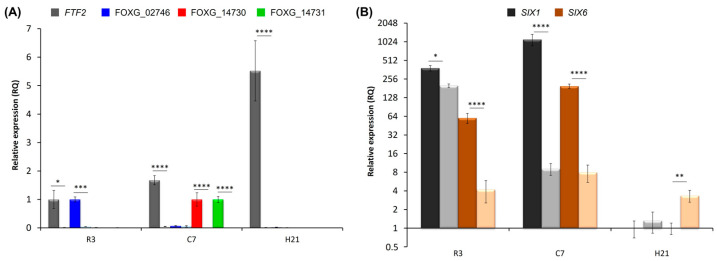
RT-qPCR analysis of the expression of putative FTF2-responsive genes (**A**) and two SIX effector genes (**B**) during common bean colonization by a FOP-SP1Δ*FTF2* mutant. The plant regions assayed and time intervals after inoculation are indicated in the *X* axis (R3, root system 3 dpi; C7, root crown 7 dpi; H21, hypocotyls 21 dpi). The relative expression measurements in the *Y* axis in (**B**) are indicated in a logarithmic scale. Dark bars indicate expression in plants colonized by FOP-SP1; light bars indicate expression in plants colonized by FOP-SP1Δ*FTF2*. The expression ratios were normalized by using the *EF1α* gene as an endogenous control. The value 1.0 was denoted for the transcript level of genes *FTF2* and FOXG_02746 in wild-type R3, of genes FOXG_14730 and FOXG_14731 in wild-type C7, and genes *SIX1* and *SIX6* in wild-type H21. The levels of expression for a pair of measurements were tested using a *t*-test and significant differences are indicated by * (*p* < 0.05), ** (*p* < 0.01), *** (*p* < 0.001), and **** (*p* < 0.0001).

Second, the expression of the selected genes was analyzed in the course of plant colonization by the wild-type and corresponding *FTF2* mutant strains. To this point, RT-qPCR experiments were performed with roots 3 days post-inoculation (dpi), root crown 7 dpi, and hypocotyls 21 dpi (Figure 8). None of the genes analyzed were expressed in a detectable way in plants inoculated with the Δ*FTF2* mutant, thus showing that expression of *FTF2*, and not of *FTF1*, is required for their in planta expression. The results presented in Figure 8A show a moderate induction of the expression of *FTF2*, which reaches a maximum in hypocotyl samples at 21 dpi. Transcripts of FOXG_02746 (the *locus* encoding a hydrophobin II) were detected in the early stages of infection, while transcripts of FOXG_14730 (the *locus* encoding a protein with SET domain) and FOXG_14731 (the *locus* encoding an oxygenase) were only detected in root crown samples. By the time vascular wilt was at its maximum and the plants were dead (21 dpi), the expression of none of the FTF2-responsive genes could be detected.

*FTF2*-overexpression mutants were obtained in *F. oxysporum* f. sp. *lycopersici*, and it has been suggested that FTF2 regulates the expression of genes encoding the SIX effectors [25]. Although none of the *SIX* genes described to date were selected as putative FTF2-responsive genes, we analyzed *SIX1* and *SIX6* expressions during colonization (Figure 8B). A significant reduction in the accumulation of both transcripts was observed in the root and root crown of plants colonized by FOP-SP1Δ*FTF2*, indicating that *SIX1* and *SIX6* are directly or indirectly regulated by FTF2. Curiously, the expression of SIX6 in hypocotyls at 21 dpi was significantly higher in plants colonized by FOP-SP1Δ*FTF2* than in those colonized by the wild-type FOP-SP1.

## 4. Discussion

An interesting advantage of accessory genomes could be the acquisition of novel virulence factors that can expand host range [26] or modify the colonizing abilities of plant pathogens. Novel or modified gene functions evolved from gene duplications and subsequent divergence might be well-tested in the context of accessory chromosomes. It is not easy to explain how this mechanism might produce entirely new proteins able to perform new functions, except that already existing transcription factors could change to alternative forms with different regulatory abilities, thus reprograming the expression of whole sets of genes. The best-known pathogenicity chromosome in *F. oxysporum* is chromosome 14 in the reference strain *F. oxysporum* f. sp. *lycopersici* 4287, which is part of the accessory genome. It contains 13 predicted transcription factor genes that cluster into nine families [25]. Interestingly, all these transcription factors, except TF3, have a homolog in the core genome, which strongly support the hypothesis that they have evolved by gene duplication. In the case of the *FTF* transcription factors gene family, the core genome homolog is *FTF2* (chromosome 9 in the reference strain), and all the *FTF1* paralogs are located in chromosomes pertaining to the accessory genome (chromosomes 3, 6, 14, 15, and the variable segment of chromosome 1) [10]. In the highly virulent strains of *F. oxysporum* f. sp. *phaseoli*, where this gene family was first described [8,11,14], all the *FTF1* paralogs are located in a small chromosome, likely the homolog to chromosome 14 [10,11,27].

To ascertain what advantages in terms of virulence may provide the FTF1 transcription factors, a comparative analysis with FTF2 is required. In a former work, we generated Δ*FTF2* mutants from a highly virulent strain (FOP-SP1) to analyze *FTF1* when *FTF2* is inactive [10]. In the present work, we described and analyzed Δ*FTF2* mutants from a weakly virulent strain (FOP-SP4), which is devoid of all the *FTF1* paralogs, with the aim to study the phenotypic effect of the complete absence of *FTF* genes.

First, multiple alignments and three-dimensional modeling were conducted to identify the differences in the predicted spatial structure of the two main domains: the Zn(II)_2_Cys_6_ binuclear cluster and middle homology region. No significative differences were observed in the former one that might account for the differences in DNA-binding capabilities. The MHR domain is involved in the regulation of the activity of Zn(II)_2_Cys_6_ binuclear transcription factors [28]. The crystal structure of Cep3, a fungal transcription factor containing an MHR region, has showed that this region contains eight motifs included in an all-alpha domain, called MHD (Middle Homology Domain) [29,30]. The multiple alignment of the amino acid sequences that comprise MHD indicates that this domain shows some diversity, both in comparison to FTF2 and among the FTF1 proteins. However, the 3D analysis did not show any evidence that those differences might be correlated with modifications of the basic all-alpha domain. It cannot be ruled out that some of the polymorphisms observed may alter the function of the corresponding FTF1 proteins. However, both the alignment and 3D modeling do not show changes in the all-alpha domain between FTF2 and all the FTF1 proteins that may suggest differences in the regulation abilities of both types of transcription factors.

Mutants altered in *FTF2* showed slight differences with the wild types in sporulation and germination rates. However, the most striking difference was the drastic increase in the production of macroconidia when grown on solid media (either PDA or minimal medium supplemented with different carbon sources). Several transcription regulators essential for conidiation were described in *F. oxysporum*. REN1 is required for the normal development of micro- and macronidia [31], while STUA is a positive regulator for the development of macroconidia and a negative regulator for the development of chlamydospores [32]. Additionally, several components of the Velvet complex are involved in conidiation. The disruption of *veA*, *velB*, and *velC* causes a derepression of conidiation and differences in the shape and size of microconidia [33]. The phenotype displayed in solid media by the *FTF2* mutants is different to those described above and strongly suggests that FTF2 might be a negative regulator of the production of macroconidia.

*FTF2* mutants show a slight reduction in virulence [10]; however, whether this is caused by an enhanced plant defensive response or a reduction in colonization capabilities has not been elucidated. The results here reported strongly indicate that both causes contribute to the observed reduction. The plant colonization pattern exhibited by the *FTF2* mutant here analyzed is characterized by first, a reduced ability to colonize the root system at early stages, and second, an increased ratio of parenchymal colonization versus vascular colonization at later stages. Both features are also distinctive of the colonization patterns exhibited by weakly virulent strains [20] and mutants attenuated in the expression of the *FTF* genes [10], although the *FTF2* mutants are more virulent than the mutants attenuated in the *FTF* genes and slightly less virulent than weakly virulent strains. The reduced colonization of the root system is not a consequence of the reduction in fungal biomass, as it was also observed for the weakly virulent strains [20]. Our results strongly support the role of the *FTF1* paralogs as critically required for the massive colonization of the vascular system, which results in enhanced virulence towards the host plant. The higher rate of parenchymal colonization displayed by the *FTF2* mutant correlates with an increased expression of *PR1* by the host plant. This result agrees with former reports on the colonization pattern showed by weakly virulent strains [20].

Taking together all these observations, two basic plant colonization patterns may be depicted. Naturally occurring weakly virulent strains (such as FOP-SP4) or mutants with an altered expression of virulence factors (such as those defective in *FTF2* or attenuated in the *FTF* genes) show an increase in the parenchymal/vascular colonization ratio that correlates with an induction of the host defensive response mediated by SA (as shown by the increased expression of *PR1*). On the contrary, highly virulent strains rapidly entered the vascular cylinder and spread through the xylem vessels of the host plant. This rapid progression towards the vasculature of the plant requires the avoidance of the SA mediated plant defensive response and high expression of all the members of the *FTF* gene family.

The structure of the functional domains in FTF2 and FTF1 do no suggest functional differences between both transcription factors. However, we show that, at least under different growing conditions, they regulate different genes. The genes encoding for hydrophobin II, the protein with a SET domain, and oxygenase did not show detectable expression during plant colonization by the FOP-SP1 mutant that lacks *FTF2*; although, they responded to *FTF1* overexpression during in culture growth. These results demonstrate that although FTF1 may potentially regulate these genes, their naturally occurring regulation, both in culture and in planta, correspond to FTF2.

Among the five genes selected to verify their possible regulation by FTF2, loci FOXG_02748 and FOXG_02746 were predicted to encode fungal hydrophobins. Hydrophobins are low-molecular-weight-secreted proteins. They are characterized by moderate to high levels of hydrophobicity and the presence of eight conserved cysteines [34]. The established role for hydrophobins in fungi is to form a layer that allows fungal structures to breach the air–water interface or prevent water-logging [35]. They are also involved in rendering the conidial surface hydrophobic and resistant to water, thus facilitating the dispersal of spores in the air [36]. Surface hydrophobins play a role in preventing the immune recognition of airborne fungal spores [37]. Hydrophobins have been shown to play a role in fungal plant pathogenicity as mutants altered in their expression show reduced virulence towards the host plant [38,39,40]. This contribution to virulence has also been confirmed in *F. graminearum*, as mutants deficient in hydrophobins FgHyd2 and FgHyd3 show reduced growth and ability to penetrate through the water–air interface in the plant host [41]. The regulation of the expression of hydrophobin-encoding genes by FTF2 suggests the involvement of this transcription factor in conidia production and also in plant colonization, in accordance with the phenotype described for null mutants. These results are consistent with those reported for another xylem colonizer, such as *Verticillium dahliae*, which is able to produce hydrophobins in tomato xylem sap [42].

Transcriptomic analysis performed for the *FTF2* overexpression mutants of *F. oxysporum* f. sp. *lycopersici* suggested that two SIX efectors (SIX1 and SIX6) were regulated by FTF2 [25]. Our results confirm this role, as both SIX1 and SIX6 are downregulated in FOP-SP1Δ*FTF2* mutants during host colonization. Previously, it was shown that SIX1 and SIX6 were under positive control of the *FTF1* paralogs [10]. The results here presented extend the positive control of SIX effectors to the whole *FTF* gene family. Taking into account the differential expression of the members of the family in the time/spatial frame of plant colonization, it is likely that a fine modulation of the expression of the effectors occurred.

**Figure 9:**
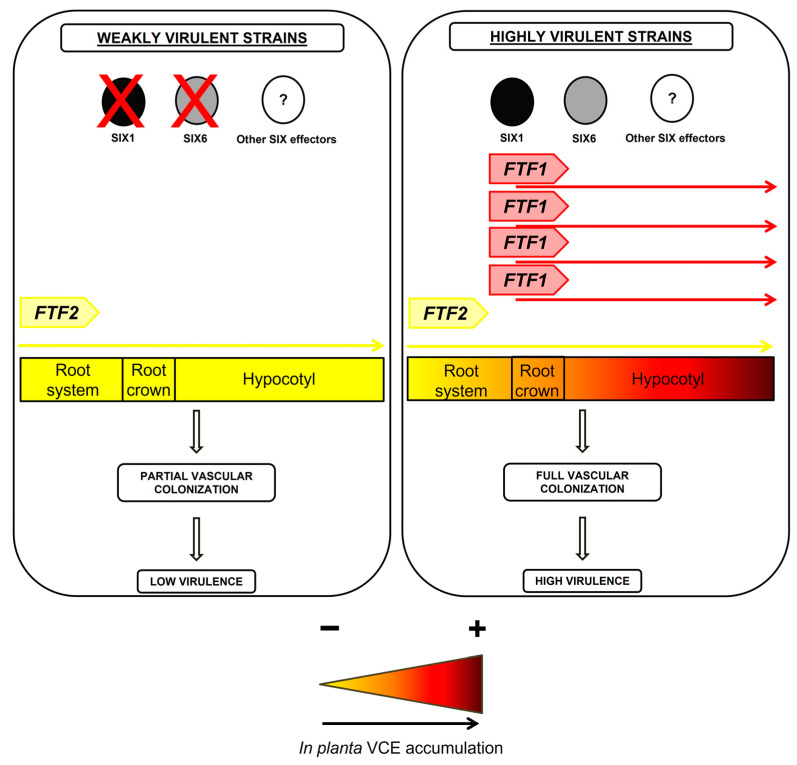
Hypothetical model of the regulation of vascular colonization effectors (VCEs) and virulence during host plant colonization by *F. oxysporum*. VCE: vascular colonization effectors. The yellow to red shade transition represents the buildup of effectors as the result of gene expression activation by the FTF family of transcription factors and the subsequent enhanced virulence.

We propose the model depicted in Figure 9 to explain the role of the FTF family of transcription factors in plant colonization and the severity of disease produced by *F. oxysporum* [27]. Virulence and the degree of symptoms produced in the host would be the consequence of a cumulative expression of the active members of the *FTF* gene family and the repertoire of effectors present in the fungal strain. The lack of *FTF1* paralogs and a reduced number of *SIX* effectors would result in mild symptoms, such as those displayed by weakly virulent strains. On the other hand, an extended set of effectors and several active copies of the *FTF1* paralogs would drastically increase the severity of disease (including the death of the plant), as it occurs in the infections produced by highly virulent strains. This model predicts the probable genetic features of *F. oxysporum* endophytes. As these strains are able to colonize plant parenchymal tissues with almost no growth inside the vasculature, they should lack *FTF1* paralogs and display a reduced effector gene repertoire. A recent study conducted on *F. oxysporum* endophytes and more than 100 pathogenic strains confirms this prediction. It was observed that a pathogenic lifestyle is associated with extended effector gene catalogs, while the endophytes analyzed clustered together based on the scarcity of effector candidates in their genomes [43]. Although no data on the presence of *FTF1* are provided in the study, all the endophytic *F. oxysporum* strains we analyzed to date were devoid of *FTF1* copies, as it occurred for the weakly virulent strains (data not shown). Moreover, the presence of *FTF1* is proof of virulence [18].

The expression of the *FTF* gene family members to regulate virulence offers a good example of the coordinated crosstalk between elements of the core and accessory genomes in *F. oxysporum*. The need for a spatial association between the gene expansion of transcription factors and their regulated genes has been suggested [44]. However, there is no association between the *FTF2 locus* (chromosome 9) and target genes *SIX1* and *SIX6* (chromosome 14), thus showing that transcription factors in the core genome may regulate effector genes in the accessory genome.

## 5. Conclusions

We provided evidence that FTF2 is a negative regulator of macroconidia production, which, together with the predicted functions inferred for some of the genes it regulates, supports its role in basic metabolism and physiology, as it would be expected for a core genome gene. FTF2 is also required for full virulence, probably in a cumulative but not redundant manner with the FTF1 transcription factors, as it is described in the model proposed for virulence regulation by the *FTF* gene family. The appearance of multiple paralogs by gene duplication and diversification of *FTF2* in the accessory genome would provide highly virulent strains with a selectable advantage in terms of the quantitative positive regulation of effectors required for the rapid colonization of xylem vessels. It remains to be clarified whether FTF2 is critical for plant colonization also in endophytic strains, or only for virulence in a pathogenic lifestyle.

## Data Availability

No new data were created or analyzed in this study. Data sharing is not applicable to this article.

## Supplementary Materials

The following supporting information can be downloaded at: https://www.mdpi.com/article/10.3390/pathogens12030380/s1, Figure S1: Schematic representation of the *FTF2* wild type allele and Δ*FTF2* mutant allele and Southern blot hybridization analysis of Δ*FTF2* mutants; Figure S2: Saprophytic growth on solid media of FOP-SP1 Δ*FTF2* and FOP-SP4 Δ*FTF2* mutant strains; Figure S3: Fusarium wilt induced in common bean plants by *Fusarium oxysporum* f. sp. phaseoli Δ*FTF2* mutants derived from the weakly virulent strain FOP-SP4; Figure S4: Quantification of fungal biomass in Phaseolus vulgaris plants colonized by FOP-SP1 Δ*FTF2* strains; Table S1: Oligonucleotides used in this work; Table S2: Quantification of macroconidia (%) produced by FOP-SP1ΔFTF2 mutants.
