# Supplementary material for: The Role of the Fusarium oxysporum FTF2 Transcription Factor in Host Colonization and Virulence in Common Bean Plants (Phaseolus vulgaris L.)
Source: Pathogens. 2023 Feb 26;12(3):380. doi: 10.3390/pathogens12030380 (PMC10054582; doi:10.3390/pathogens12030380)
Supplement: Supplementary file 1 [file pathogens-12-00380-s001.zip › pathogens-2183354-supplementary.pdf]

Supporting information

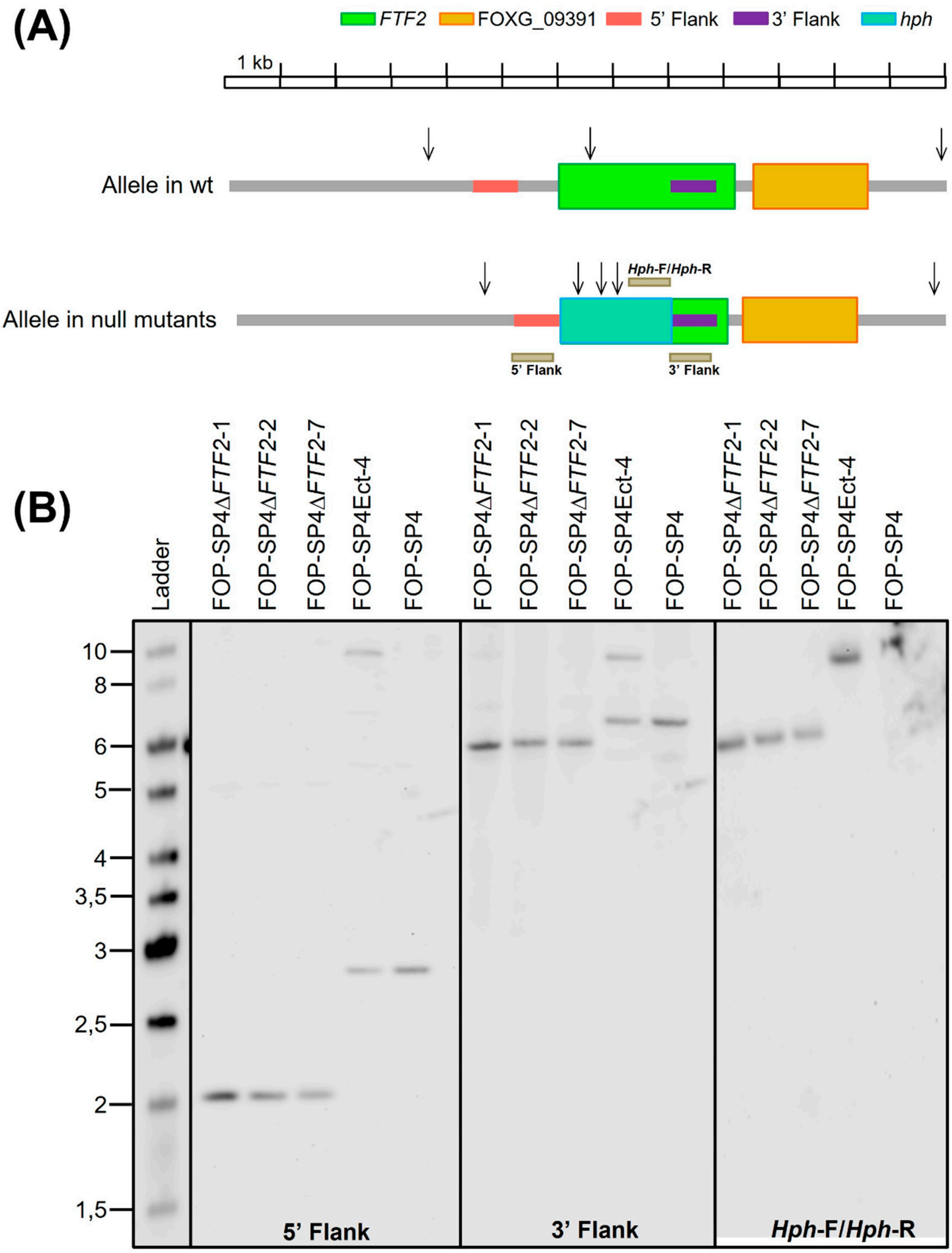

Supplementary Figure S1. (**A**) Schematic representation of the recognition sites of *Bam*HI (black arrows) in the wild-type allele of *FTF2* and the allele in the null mutants. Brown boxes show the location of the probes used in the Southern blot analysis. (**B**) Southern blot hybridization analysis of D*FTF2* mutants in FOP-SP4 strain. *Bam*HI-digested DNA from from mutants FOP-SP4Δ*FTF2*-1, FOP-SP4Δ*FTF2*-2, FOP-SP4Δ*FTF2*-7 and FOP-SP4Ect-4 and from wild-type strain FOP-SP4 was hybridized with a probe corresponding to the 5′ flanking region (probe 5′ Flank), a probe corresponding to the 3′ flanking region (probe 3′ Flank) and a probe derived from the hph ORF (probe *Hph*-F/*Hph*-R). 'Ladder' identifies the molecular marker 1 kb DNA ladder (Biotools), sizes are shown in kb.

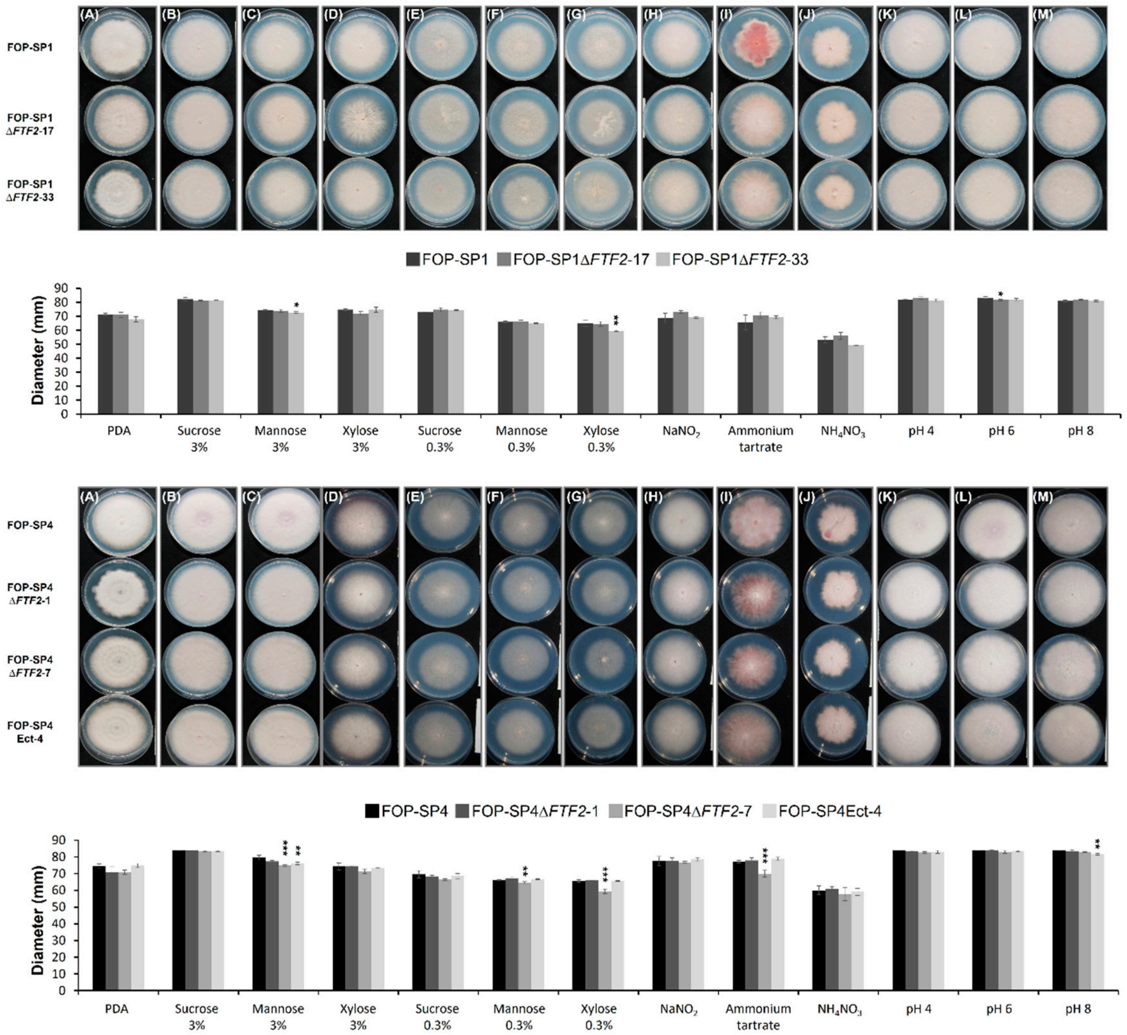

Supplementary Figure S2. *S*aprophytic growth on solid media of FOP-SP1Δ*FTF2* and FOP-SP4Δ*FTF2* mutant strains. For each media, colony diameter was determined 6 days post-inoculation. Bars represent the media ± standard deviation of 3 independent biological experiments. Differences between mutant and control strains were test with an ANOVA analysis followed by a Dunnett's test and are indicated as * ($P < 0.05$), ** ($P < 0.01$) and *** ($P < 0.001$). Colony morphology and pigmentation were tested 6 dpi on PDA (**A**), on minimal media amended with 3% sucrose (**B**), 3% mannose (**C**), 3% xylose (**D**), 0.3% sucrose (**E**), 0.3% mannose (**F**) or 0.3% xylose (**G**), on minimal media amended with 3% sucrose and $NaNO_2$ (**H**), ammonium tartrate (**I**) or NH_4NO_3 (**J**), and on minimal media amended with $NaNO_3$ and 3% sucrose at pH 4 (**K**), pH 6 (**L**) or pH 8 (**M**).

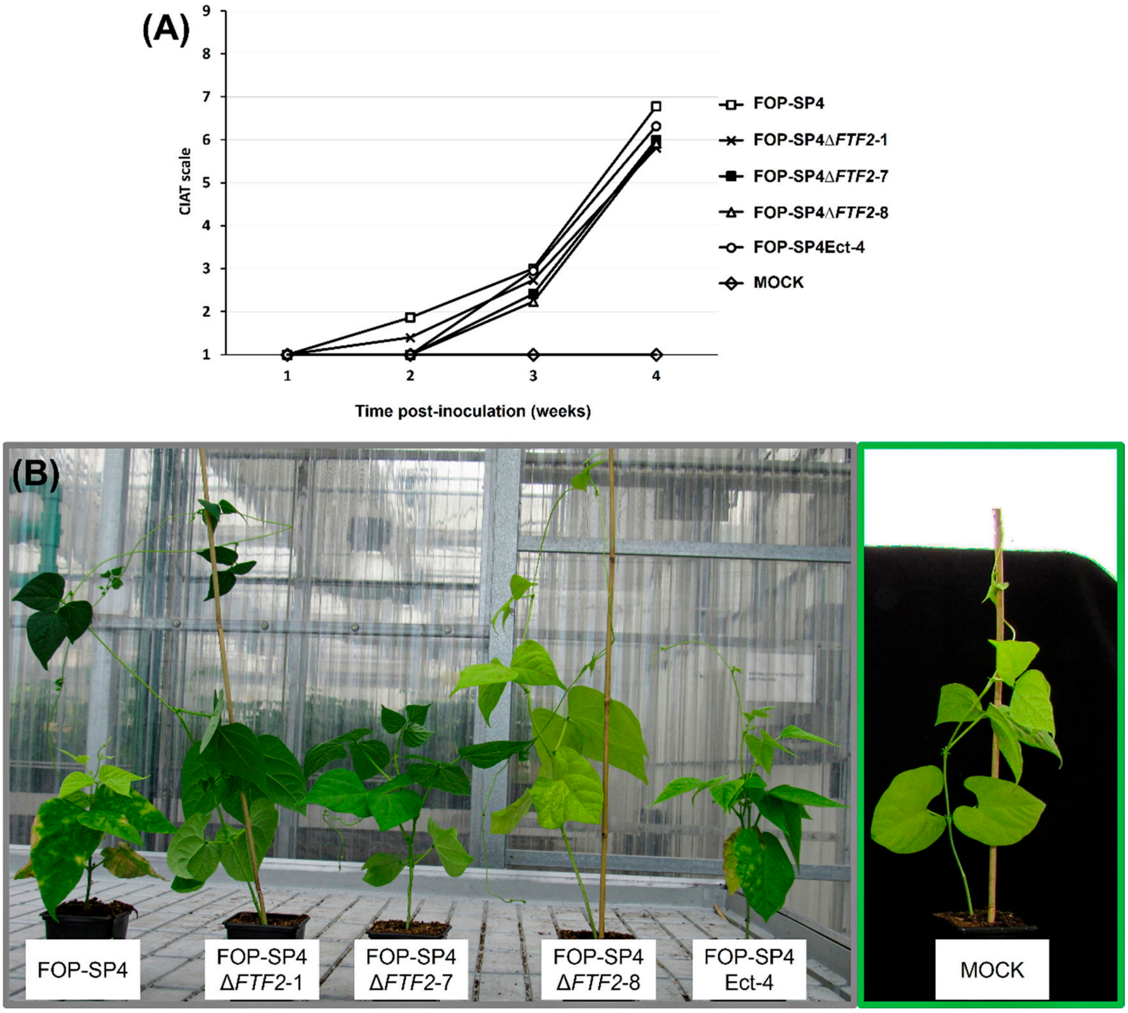

Supplementary Figure S3. Fusarium wilt induced in common bean plants by *Fusarium oxysporum* f. sp. *phaseoli* D*FTF2* mutants derived from the weakly virulent wild type FOP-SP4. (A) Severity of Fusarium wilt disease symptoms measured by the CIAT (International Center for Tropical Agriculture) scale (the disease index is assigned according to the percentage of chlorotic or necrotic leaves). (B) Common bean plants 4 weeks after inoculation with three different *FTF2* null mutants derived from FOP-SP4: FOP-SP4Δ*FTF2*-1, FOP-SP4Δ*FTF2*-7 and FOP-SP4Δ*FTF2*-8 and ectopic transformant obtained with the gene replacement construction (FOP-SP4Ect-4).

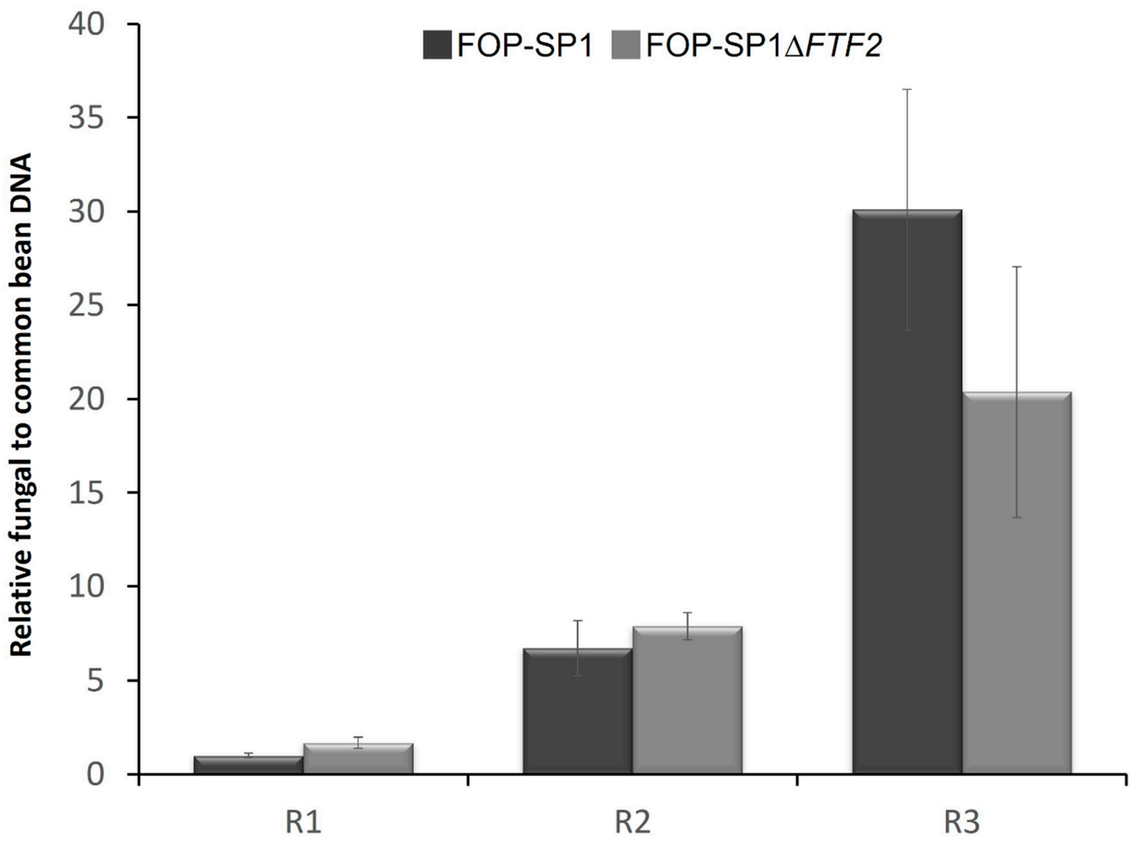

Supplementary Figure S4. Quantification of fungal biomass in *Phaseolus vulgaris* plants colonized by FOP-SP1Δ*FTF2* strains. Fungal DNA relative to that of common bean was measured by assaying the fungal *SGE1* gene and the plant *PR1* gene by RT-qPCR using DNA extracted from the root system at 1, 2 and 3 dpi (R1, R2 and R3). All measurements were referred to the value obtained for FOP-SP1 colonization at R1 (arbitrary value of 1.0).

Table S1. Oligonucleotides used in this work.

Oligonucleotide	Accesion code or plasmid	Sequence (5′-3′)
**InacFTF2-O1**	FOXG_09390[a]	GGTCTTAAUGACAGCGACAACCCAAGCCAAAC
**InacFTF2-O2**	FOXG_09390[a]	GGCATTAAUCCCTGCCCTGCGAGAGAGACAAG
**InacFTF2-A3**	FOXG_09390[a]	GGACTTAAUATGAAGCCAACAGCGGAAGCC
**InacFTF2-A4**	FOXG_09390[a]	GGGTTTAAUATATCCTGACCACCACCCAACCC
***Hph*-F**	pRF-HU[d]	GCGCTTCTGCGGGCGATTTG
***Hph*-R**	pRF-HU[d]	CGGGTTCGGCCCATTCGGAC
***EF1alpha*-Fwd**	FOXG_03515[a]	CATCGGCCACGTCGACTCT
***EF1alpha*-Rev**	FOXG_03515[a]	AGAACCCAGGCGTACTTGAA
**Int-q*FTF2*-Fwd**	FOXG_09390[a]	CGCTGTGCCATCTCCCTCTCA

**Int-q*FTF2*-Rev**	FOXG_09390[a]	CGTGGGCGTGGGCGTGAT
***SIX1*-Fwd**	AJ608702[b]	GAGCCGCCTCAATCGCCTG
***SIX1*-Rev**	AJ608702[b]	GGCCAAGTTGCGCGATATGTG
***SIX6*-Fwd**	ACY39286,1[b]	GCTTTTGCGTGGCGAACCC
***SIX6*-Rev**	ACY39286,1[b]	TTTTCCCGTTGCTGAGATTGCG
SGE1-Fwd	FOXG_10510[a]	CAGCCGTATCCTTGGCAACTA
***SGE1*-Rev**	FOXG_10510[a]	TGGTTGACTTGCCGTTCCTT
**14731-Fwd**	FOXG_14731[a]	TCGTCATATCGCCAGGGGTG
**14731-Rev**	FOXG_14731[a]	CAACGTATCGTGAGCCCCGA
**02748-Fwd**	FOXG_02748[a]	GATCCTTGCCCCGACAGTCT
**02748-Rev**	FOXG_02748[a]	GCGCAGATGGCTTGGAAGTT
**02746-Fwd**	FOXG_02746[a]	TCTTCGGAAACCCCCAGTGC
**02746-Rev**	FOXG_02746[a]	CCTTCTGGCCAGACTCGGC
**14730-Fwd**	FOXG_14730[a]	CGTCATATCGCCAGGGGTGA
**14730-Rev**	FOXG_14730[a]	TCAACGTATCGTGAGCCCCG
**04430-Fwd**	FOXG_04430[a]	CCAAGACCGCAAGGGTAACATGCT
**04430-Rev**	FOXG_04430[a]	TCGAACTTGAAGGGATAGGATGTCGC
***Actin*-Fwd**	Phvul.011G064500[c]	GAAGTTCTCTTCCAACCATCC
***Actin*-Rev**	Phvul.011G064500[c]	TTTCCTTGCTCATTCTGTCCG
***ERF1*-Fwd**	Phvul.007G127800[c]	CCTGTTGGGCTCTGAAGAGGAAAC
***ERF1*-Rev**	Phvul.007G127800[c]	AGGACCAAGGTCTTCAAACACGAC
***ERF2*-Fwd**	XM_003549886[d]	GGGAAAGTTCGCGGCGGAG
***ERF2*-Rev**	XM_003549886[d]	CGGAGTTAACCCTCAACGGAAAATTC
***PR1*-Fwd**	Phvul.003G109100[c]	CAGGCACTACACTCAGGTTGTTTGGA
***PR1*-Rev**	Phvul.003G109100[c]	TTGCCAGGAGGAGCATAGTTGCAA

(a) *Locus* in the genome of the strain 4287 of *F. oxysporum* f. sp. *lycopersici*; (b) *GenBank* accession number; (c) *locus* in the genome of *P. vulgaris*; (d) *locus* in the genome of *Glicine max*.

Table S2. Quantification of macroconidia (%) produced by FOP-SP1Δ*FTF2* strains.

	**FOP-SP1**	**FOP-SP1Δ*FTF*2-17**	**FOP-SP1Δ*FTF*2-33**
**PDA**	0	21.63 ± 15.2 ***	22.66 ± 5 ***
**Glucose**	0	78.07 ± 6.4 ***	91.74 ±8.9 ***
**Sucrose**	0	81.31 ± 10.9 ***	84.72 ± 9.6 ***
**Xylose**	0	72.8 ± 8 ***	80.48 ± 8.8 ***
**Mannose**	0	75.59 ± 5.4 ***	67.28 ± 6.4 ***
**Glycerol**	0	81.81 ± 16.2 ***	84.63 ± 18.7 ***

Numbers show the percentage of macroconidia over the total harvested conidia per condition. The data represent the media ± standard deviation of three independent biological experiments; differences were tested using an ANOVA analysis followed by a Dunnett's test and are indicated by *** ($P < 0.001$).
